# RAB7B as a Potential Therapeutic Target in Liver Cirrhosis: Insights from Protein Expression and Bioinformatics Analyses

**DOI:** 10.1021/acsomega.5c08027

**Published:** 2025-12-01

**Authors:** Jinyao Dai, Shuaibing Ying, Jie Lin, Yong Pan, Qiudan Zhang, Jing Liu, Yan Lou, Yunqing Qiu

**Affiliations:** † State Key Laboratory for Diagnosis and Treatment of Infectious Diseases, National Clinical Research Center for Infectious Diseases, National Medical Center for Infectious Diseases, Collaborative Innovation Center for Diagnosis and Treatment of Infectious Disease, Zhejiang Provincial Key Laboratory for Drug Evaluation and Clinical Research of Zhejiang Province, The First Affiliated Hospital, 71069 Zhejiang University School of Medicine, Hangzhou 310003, China; ‡ Department of General Surgery, Sir Run-Run Shaw Hospital, Zhejiang University School of Medicine, Hangzhou 310003, China

## Abstract

Liver cirrhosis remains a major global health challenge, with liver transplantation currently representing the most effective treatment. Elucidating its molecular pathogenesis is therefore critical for the development of novel therapeutic strategies. Emerging evidence indicates that mitophagy plays a pivotal role in cirrhosis progression. In this study, we employed integrative bioinformatics to explore the association between mitophagy and liver cirrhosis, aiming to identify potential therapeutic targets. Differentially expressed genes (DEGs) were obtained from GSE77627 and GSE139602 data sets, followed by functional enrichment analysis. Mitophagy-related differentially expressed genes (mito-DEGs) were screened and assessed through receiver operating characteristic (ROC) analysis, immune cell infiltration profiling, and protein–protein interaction (PPI). Weighted gene coexpression network analysis (WGCNA) identified key modules, and intersecting genes with mito-DEGs highlighted RAB7B as a candidate hub gene, which was validated in external data sets. Experimental verification confirmed elevated RAB7B expression in activated hepatic stellate cells (HSCs) and a mouse model of liver cirrhosis. Functional assays demonstrated that RAB7B inhibition attenuated HSC activation, and molecular docking revealed strong binding affinity between RAB7B and predicted anticirrhosis compounds. We found that RAB7B knockdown restored TGF-β-induced mitophagy inhibition, enhanced mitochondria-lysosome colocalization, and showed dynamic regulation by mitophagy status, indicating its role as a negative and responsive regulator of mitochondrial clearance. Collectively, our findings identify RAB7B as a mitophagy-related hub gene driving liver cirrhosis progression and provide novel insights into its therapeutic potential.

## Graphical abstract

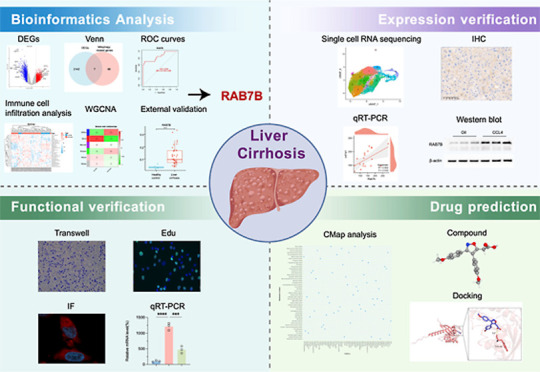

## 1 Introduction

Liver cirrhosis arises from chronic liver conditions, including viral hepatitis, alcoholic liver disease, and metabolic dysfunction-associated fatty liver disease (MAFLD). It is defined by diffuse fibrosis, structural destruction of liver tissue, and the formation of regenerative nodules.^1–3^ Hepatocellular carcinoma has long been associated with liver cirrhosis, which is also a major cause of liver failure morbidity and death.^4^ The 2021 Global Burden of Disease Study showed an age-standardized prevalence rate of 20,302.6 per 100,000 population for liver cirrhosis, with sustained increases in prevalence and incidence rates.^5^ Currently, targeting the underlying etiology of liver cirrhosis remains the main therapeutic strategy. Nevertheless, etiologic treatments have limited efficacy in patients with advanced liver cirrhosis. Liver transplantation remains the optimal treatment for end-stage cirrhosis, however, limited by donor shortages, surgical complexity, the risk of immune rejection, and high costs.^6^ Thus, comprehensive investigations into the cellular and molecular mechanisms driving liver cirrhosis progression are urgently needed to identify novel therapeutic targets.

Mitochondria are critical organelles that maintain cellular homeostasis. They participate in a variety of processes, including signaling, biosynthesis, bioenergetics, and dynamics.^7^ Mitophagy is a mitochondrial quality control mechanism that selectively eliminates damaged mitochondria to preserve cellular homeostasis.^8,9^ Accumulating evidence establishes impaired mitophagy as a pathogenic contributor to liver disease progression.^10^ Notably, mitophagy is crucial in cirrhosis development.^11,12^ By promoting mitophagy, mitochondria-targeted antioxidants have been shown to reduce liver fibrosis in animal models of hepatic cirrhosis.^13^ Mitophagy in hepatocytes inhibits reactive oxygen species (ROS) production and inflammatory response, thereby attenuating hepatic fibrosis.^14,15^ In HSCs, inhibition of mitophagy has been observed to aggravate liver fibrosis.^16^ Consequently, mitophagy has a dual effect on liver cirrhosis, either promoting or inhibiting its progression. However, the regulatory network linking mitophagy to liver cirrhosis pathogenesis remains largely undefined, and the key mitophagy-associated genes driving fibrotic progression have not been systematically identified.

To bridge this knowledge gap, we obtained liver cirrhosis-related expression from the Gene Expression Omnibus (GEO). We identified DEGs through differential expression analysis and functionally enriched them using the Kyoto Encyclopedia of Genes and Genomes (KEGG) and Gene Ontology (GO). Subsequently, we intersected mitophagy-related genes from the KEGG and Reactome databases, and identified seven mito-DEGs by ROC analysis, immune cell infiltration analysis, and PPI networks, aiming to explore the relationship between mitophagy and liver cirrhosis. Consensus genes between mito-DEGs and WGCNA-derived module genes were analyzed to identify core regulators. Through these analyses, we identified RAB7B as a previously unrecognized regulator of mitophagy and a potential therapeutic target for liver cirrhosis. Our investigation revealed that RAB7B expression was significantly increased in activated HSCs and RAB7B knockdown showed antifibrotic efficacy in vitro experiments. These findings were substantiated in carbon tetrachloride (CCL_4_) induced cirrhotic mouse models. Additionally, the predicted antiliver cirrhosis compounds demonstrated high binding affinity toward the RAB7B. Our results demonstrated that RAB7B deficiency alleviates TGF-β-induced mitophagy impairment, promotes mitochondria-lysosome colocalization, supporting its function as a negative regulator of mitochondrial quality control. Collectively, our study uncovers a novel mechanistic link between mitophagy dysfunction and liver cirrhosis progression, highlighting RAB7B as a promising target for therapeutic intervention.

## 2 Materials and Methods

### 2.1 Data Collection

From the GEO database, the microarray data sets (GSE77627 and GSE139602) were retrieved.^17^ GSE77627 contained 22 liver cirrhosis samples and 14 healthy samples, while GSE139602 included 20 cirrhotic samples and 6 healthy samples. The two data sets were merged to generate a completely new data set of liver cirrhosis (LC data set) by the R packages “Limma” and “sva”. In addition, 75 mitophagy-related genes in Table S1 were curated from the KEGG and Reactome databases.

### 2.2 Differential Expression Analysis

The R package “Limma” was used to find DEGs between cirrhotic liver samples and healthy controls in the LC data set. Differential expression requires an adjusted *p*-value (adj. *p*) < 0.05 and an absolute value of log2 (fold change) (log2FC) > 1. The R package “ggplot2” for volcano plots^18^ and “pheatmap” for heatmap representation were used to visualize DEGs.

### 2.3 Functional Enrichment Analysis

The R package “clusterProfiler” was used to conduct GO and KEGG analyses to investigate the biological processes and functions associated with DEGs. The threshold for enrichment significance was adj. *p* < 0.05.

### 2.4 PPI Networks

The STRING database and GeneMANIA software were used to build PPI networks. The GeneMANIA database offers functional bioinformatics tools, including analysis of physical interactions, gene enrichment, localization and coexpression analysis, and subcellular prediction. To measure functional gene relationships, the STRING database combines information from several sources, including protein–protein interactions, coexpression, gene neighborhood, and fusion.^19^

### 2.5 Analysis of Immune Cell Infiltration

Immune cell infiltration quantification was performed via CIBERSORT, assessing 28 immune subsets in the LC data set. A deconvolution algorithm called CIBERSORT was utilized to describe the makeup of immune cells in a tissue.^20^ The Wilcoxon test was used to assess statistical significance (*p* < 0.05). The R package “heatmap” and “vioplot” tools were used to depict the differences in immune cell infiltration. To confirm the link between genes and immunological infiltration, Spearman’s correlation analysis was also carried out.^21^ The R tool “corrplot” was used to show the results.

### 2.6 WGCNA

WGCNA investigates the connection between gene networks and illnesses and finds gene modules with significant biological relevance.^22^ The LC data set’s disease-related modules were found by the R package “WGCNA”. The R package “VennDiagram” program was used to find shared genes between module genes and mito-DEGs.

### 2.7 Single-Cell RNA Sequencing Analysis

The single-cell RNA sequencing data set GSE137720 was analyzed via the scLiverDB.^23^ The expression of RAB7B at the single-cell level was analyzed.

### 2.8 Animal Model Preparation

Eight-week-old male C57BL/6 mice weighing between 17 and 23 g were obtained from Hangzhou GemPharmatech Co., Ltd. and were randomized to either the liver cirrhosis group or the normal control group. Every animal experiment was carried out in compliance with institutional policies and authorized by Zhejiang University’s Animal Care and Use Committee. All animal studies were conducted in full compliance with the ARRIVE guidelines 2.0. CCl_4_-induced liver cirrhosis model using the previously reported technique.^24^ In short, mice received twice weekly intraperitoneal injections of sterile CCl_4_ (1:3, dissolved in corn oil) for 6 weeks. After 48 h postfinal injection, the animals were anesthetized under isoflurane, and euthanized by cervical dislocation. Liver tissues were extracted for histological analysis.

### 2.9 Histopathology

Tissue samples were fixed with a 4% paraformaldehyde, embedded in paraffin. Histopathological changes were assessed using Masson staining, Sirius red, and immunohistochemistry.

### 2.10 Cell Culture

LX-2 cells were purchased from Wuhan Pricella Biotechnology Co., Ltd. Cells were cultured in DMEM (Gibco, USA) with an addition of 2% fetal bovine serum (FBS). Cells were cultured at 37 °C with 5% CO_2_.

### 2.11 Small Interfering RNA Transfection

Following the manufacturer’s instructions, Lipofectamine3000 (Invitrogen, USA) was used to transfect cells with either RAB7B-targeting siRNA (si-RAB7B) or negative control siRNA (si-NC). Table S2 displays the sequences of every RNA interference product.

### 2.12 Cell Migration Assay

Standard transwell assays were used to conduct the cell migration assay.^25^ For quantitative analysis, three randomly selected fields of vision were photographed. ImageJ was utilized to quantify the quantity of migrating cells.

### 2.13 EdU Cell Proliferation Assay

Under the manufacturer’s instructions, the EdU Cell Proliferation Kit (Beyotime, China) was utilized to assess cell proliferation. Using fluorescence microscopy, pictures were taken.

### 2.14 Immunofluorescence

To put it simply, cells were blocked and fixed before being treated with α-SMA antibody and a secondary antibody conjugated to AF594. Nuclear counterstaining was performed with DAPI. Images were acquired using confocal microscopy (Zeiss LSM900, Germany).

### 2.15 RNA Isolation and qRT-PCR

The Quick RNA Extraction Kit (Accurate Biology, China) was used to extract total RNA following the manufacturer’s instructions. The 2^‑ΔΔCt^ technique was used to quantify the data with β-actin as the reference gene. Table S3 lists the primer sequences that were employed.

### 2.16 Western Blot

Western blot assays were carried out as described in prior research. A 20 μg protein sample was separated by SDS-PAGE followed by Western blot. Table S4 details the primary antibodies utilized in the experiment.

### 2.17 Screening of Small-Molecule Compounds and Molecular Docking Analysis

The Connectivity Map (CMap) database is a resource that leverages differential gene expression profiles to predict potential therapeutic compounds.^26^ The top compounds were chosen based on their highest absolute connectivity scores (|score| ≥ 95). The 3D structure of the target protein was retrieved from the PDB. The protein structure was further processed using PyMOL software to remove water molecules and extract the target protein. Protein–protein docking was then performed with the GRAMM.^27^ Subsequently, LigPlot+ software was employed to analyze the chemical bonds involved in the docking results, and PyMOL was used for visualization.

### 2.18 Statistical Analyses

The data were statistically analyzed using SPSS 22.0 (SPSS Inc.) and GraphPad Prism 10 (GraphPad, USA) software. Pearson and Spearman correlation analyses were used to determine the relationship between metrics expression in cirrhosis data sets and liver fibrosis. Continuous data was analyzed between groups using two-tailed Student’s *t*-tests or one-way ANOVA, with mean ± standard deviation. *P*-values <0.05 were considered statistically significant.

## 3 Results

### 3.1 Screening of DEGs in Liver Cirrhosis and Functional Enrichment Analysis

The human LC data set (GSE77627 and GSE139602) were merged and batch effects were removed. Figure 1 illustrates the data sets and flowcharts used in this study. Distribution boxplots and principal component analysis revealed the successful elimination of batch effects (Figure S1A–D). Comparative analysis identified 2149 DEGs (|log2 FC| > 1, *p* < 0.05) between cirrhotic and healthy samples. This included 1044 upregulated and 1105 downregulated genes (Figure 2A). Heatmaps showing the expression patterns of these DEGs in liver cirrhosis and control samples are displayed (Figure 2B). The KEGG enrichment analysis found considerable enrichment in pathways associated with MAFLD and bile secretion (Figure 2C). GO enrichment analysis highlighted significant enrichment in process related to fatty acid metabolic processes, mitochondrial matrix, electron transport chain activity and oxidoreductase (Figure 2D–F).

**Figure 1:**
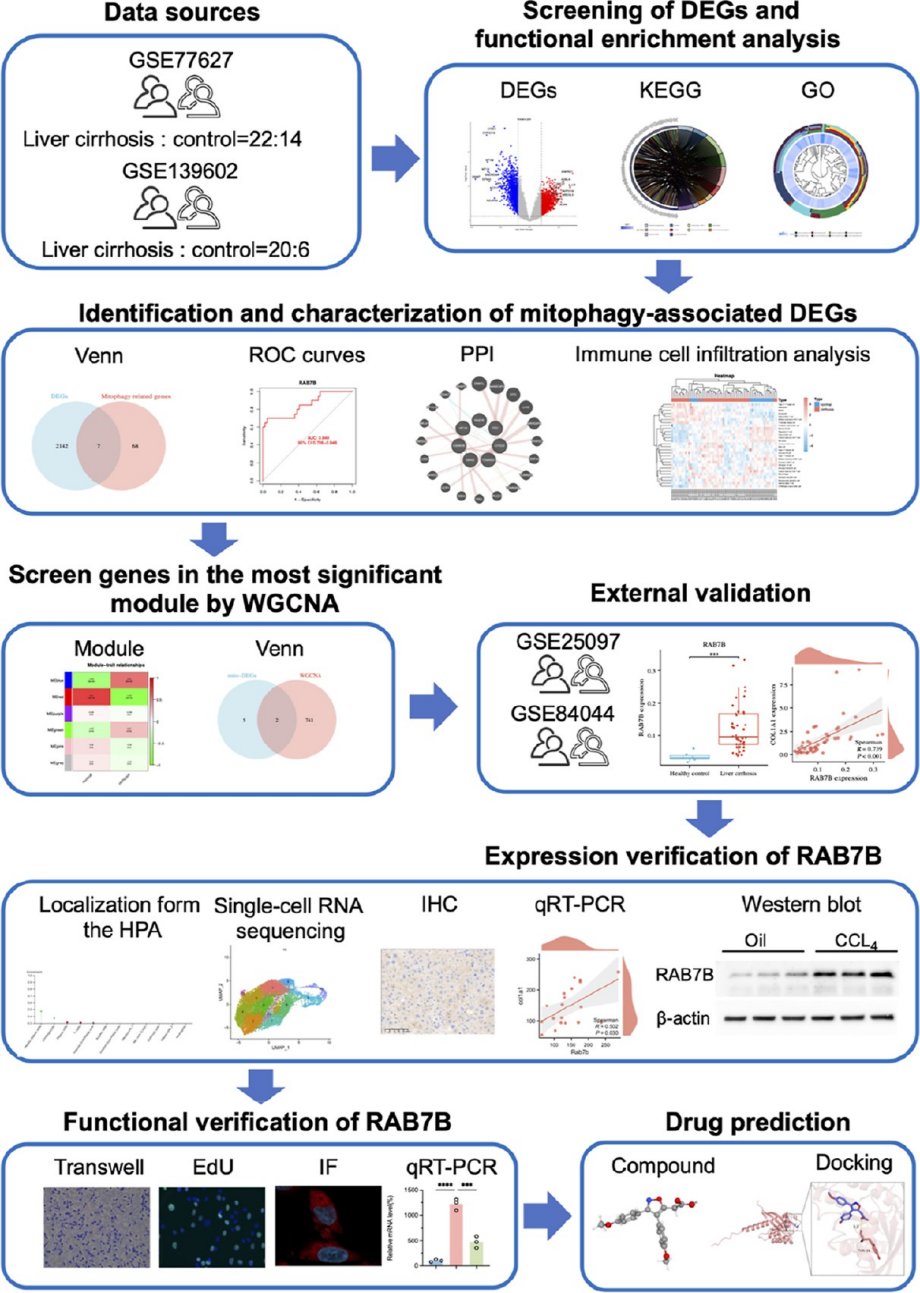
Flowchart of the analysis process.

**Figure 2:**
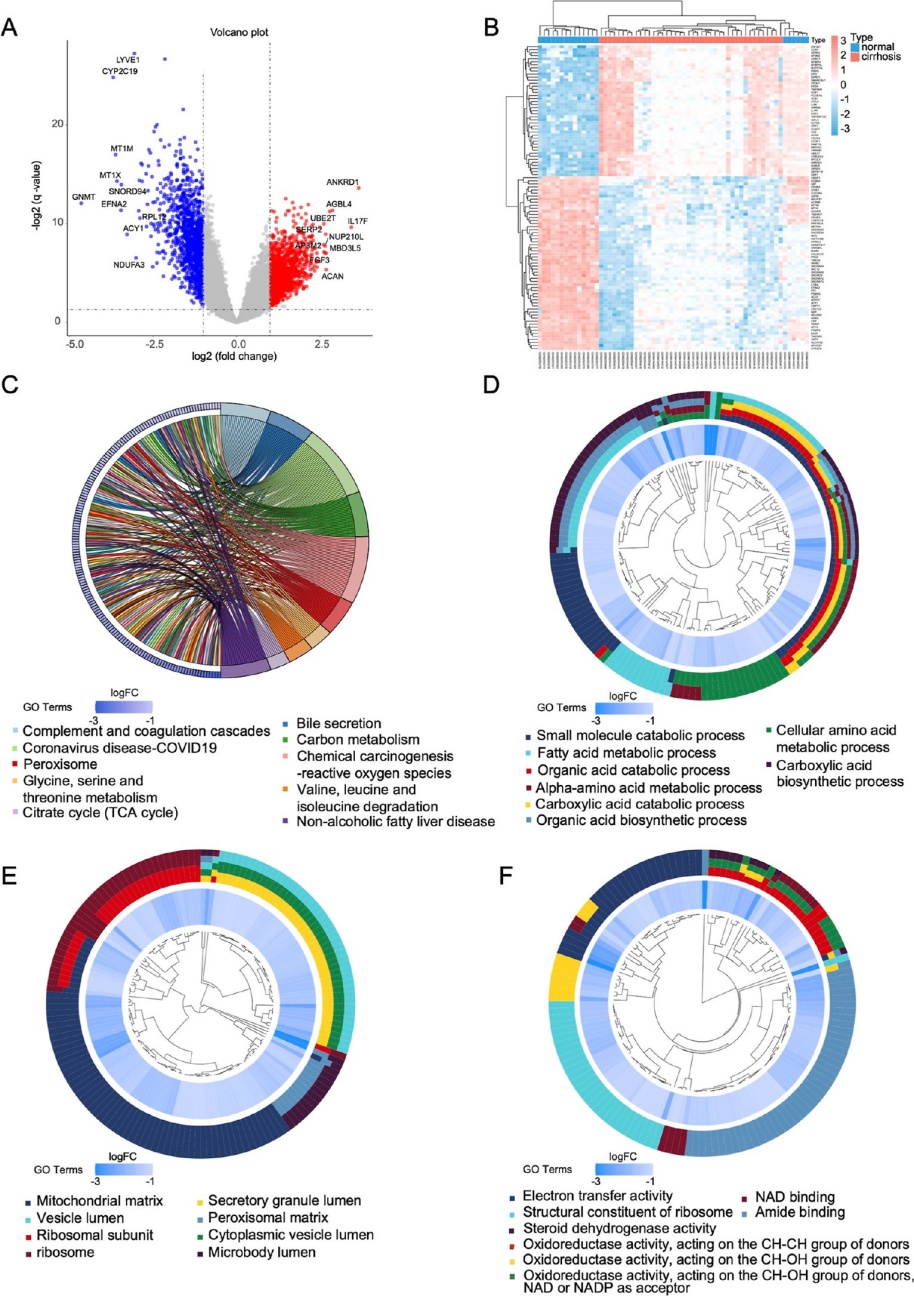
Screening of DEGs in liver cirrhosis and functional enrichment analysis of DEGs. (A) Volcano plot of LC data set. Upregulated genes are marked in red and downregulated genes are marked in blue. (B) Heatmap of LC data set (red = upregulated, blue = downregulated). (C) KEGG enrichment analysis results. The right side of the circle shows KEGG pathways with different colors, annotated below the circles. The left side displays the DEGs corresponding to the KEGG pathways on the right. (D–F) GO enrichment showing biological process (D), cellular components (E), and molecular functions (F)­analysis results. Adjusted *p* value <0.05 was considered significant.

### 3.2 Identification and Characterization of Mito-DEGs

These DEGs were intersected with mitophagy-related genes, and finally, seven mito-DEGs (NRAS, TOMM20, RAB7B, HIF1A, CITED2, FIS1, and CSNK2B) were identified (Figure 3A). Among them, NRAS, TOMM20, HIF1A, CITED2, FIS1, and CSNK2B were down-regulated and RAB7B was up-regulated in cirrhotic tissues relative to normal tissues (Figure 3B). Further, correlation analysis of these seven mito-DEGs revealed a strong positive correlation between NRAS, TOMM20, HIF1A, CITED2, FIS1, and CSNK2B, while a strong negative correlation was observed with RAB7B (Figure 3C). In addition, ROC analysis was performed for all seven mito-DEGs. The results indicated that all seven mito-DEGs yielded ROC curves with an AUC of greater than 0.7, suggesting a high accuracy in predicting liver cirrhosis formation (Figure 3D–J). GeneMANIA was used to predict 20 probable transcriptional coregulators of these seven key genes with PPI network construction of 27 total nodes (Figure 3K). Enrichment analysis about important genes and putative coregulators was conducted using the STRING database. GO enrichment analyses were primarily associated with mitochondrial protein localization, mitochondrial function, ATP metabolism, and mitochondrial outer membrane (Figure 3L). Additionally, KEGG enrichment analysis displayed that each of these genes was involved in mitophagy in animals (Figure 3M).

**Figure 3:**
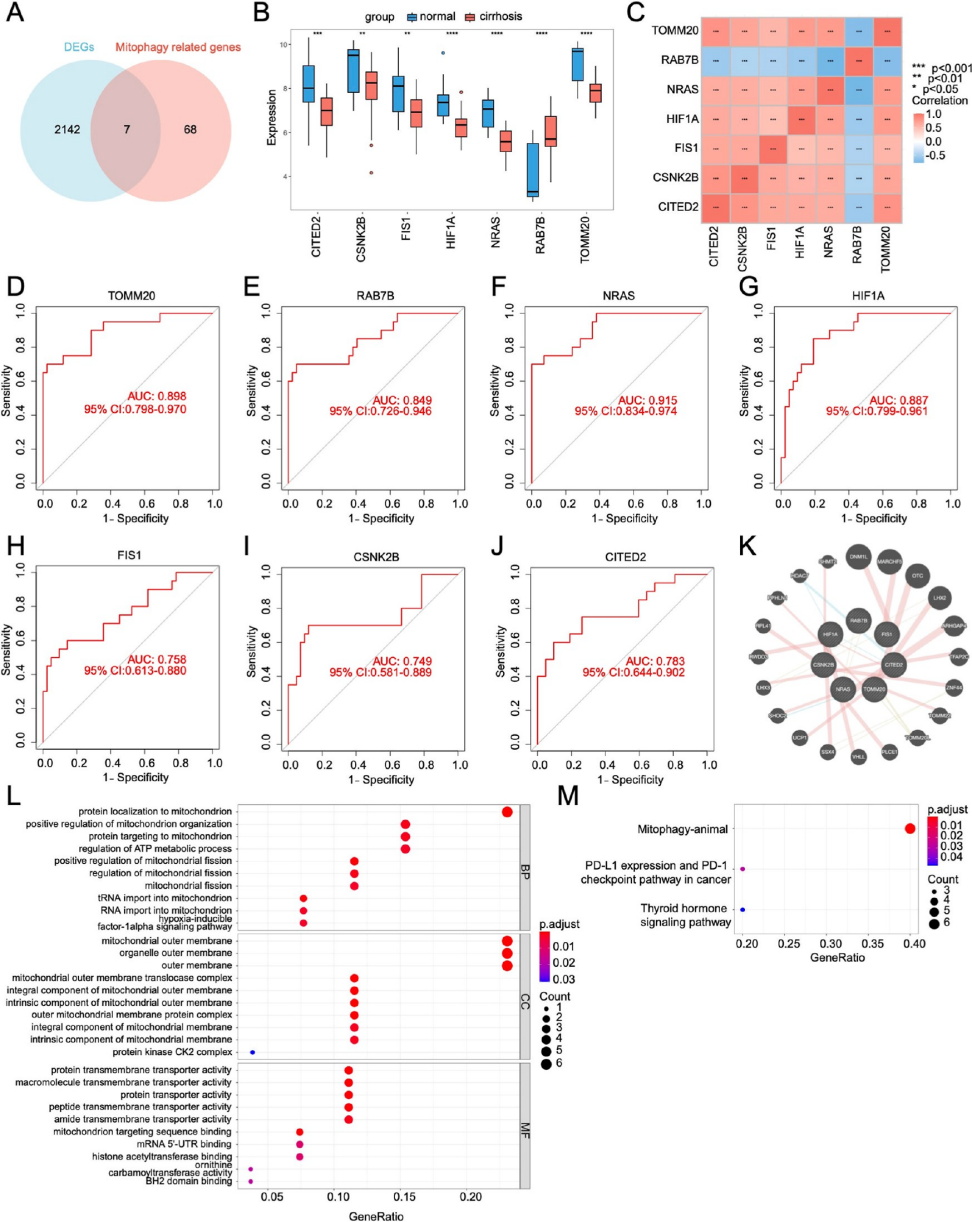
Identification and characterization of mito-DEGs. (A) Venn diagram showing genes identified from the intersection of DEGs and mitophagy-related genes. (B) Mito-DEGs expression levels in the LC data set. (C) Heatmap of correlation between mito-DEGs. (D) ROC analysis of TOMM20. (E) ROC analysis of RAB7B. (F) ROC analysis of NRAS. (G) ROC analysis of HIF1A. (H) ROC analysis of FIS1. (I) ROC analysis of CSNK2B. (J) ROC analysis of CITED2. (K) PPI network of mito-DEGs. (L,M) Functional annotation and pathway enrichment of mito-DEGs. *p < 0.05; **p < 0.01; ***p < 0.001; ****p < 0.0001.

### 3.3 Immune Cell Infiltration Analysis

To analyze differences in the immune microenvironment, we further examined the infiltration of 28 immune cell types to completely comprehend liver cirrhosis and immune cells by the CIBERSORT algorithm. (Figure 4A). Distinct immune cell distribution patterns emerged between cirrhotic and control groups, with significant intercellular correlations (Figure 4B,C). Elevated infiltration of activated CD4+ T, eosinophil, immature dendritic cell, MDSCs, and natural killer T cell was observed in the liver cirrhosis. Conversely, patients with cirrhosis had reduced numbers of effector memory CD8+ T, central memory CD8+ T, macrophage, memory B cell, monocyte, natural killer cell, and regulatory T cell cells. Significant gene–immune correlations were identified for all seven mitophagy-DEGs. The results indicated that CITED2 and RAB7B were most strongly correlated with natural killer T cells (*r* = −0.741 and 0.750, *p* < 0.001 and *p* < 0.001, respectively) and memory B cells (*r* = 0.689 and −0.724, *p* < 0.001 and *p* < 0.001, respectively); CSNK2B exhibited the strongest association with monocyte (*r* = 0.753, *p* < 0.001); FIS1 and NRAS were the most strongly correlated with memory B cell (*r* = 0.647 and 0.786, *p* < 0.001 and *p* < 0.001, respectively); and HIF1A and TOMM20 displayed the highest correlation with regulatory T cells (r = 0.773 and 0.779, *p* < 0.001 and *p* < 0.001, respectively) (Figure 4D). These results establish functional crosstalk between mitophagy regulators and immune microenvironment in cirrhosis pathogenesis.

**Figure 4:**
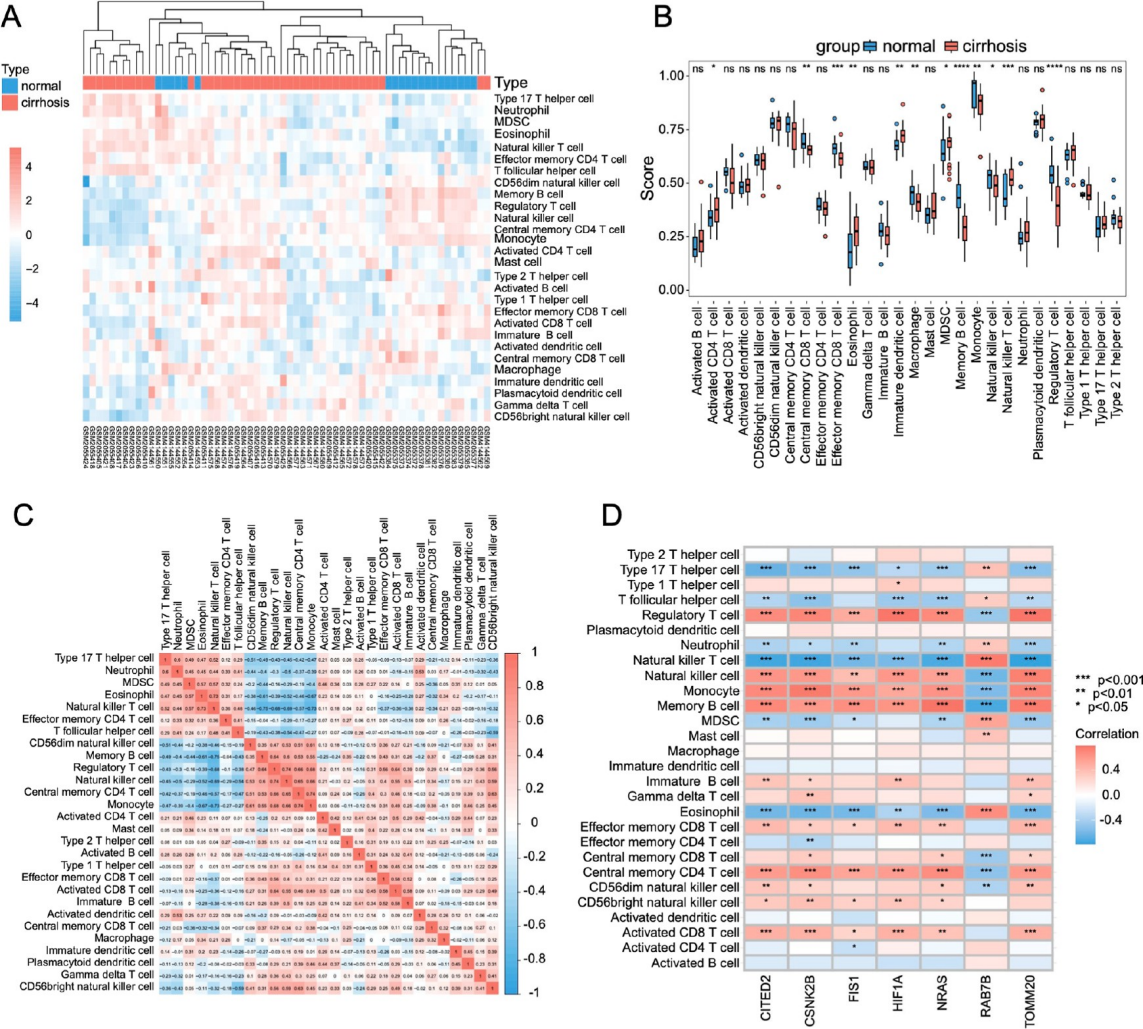
Immune cell infiltration analysis. (A) CIBERSORT-based heatmap of immune cell infiltration in the normal group (blue) and the liver cirrhosis group (red). (B) Differential analysis of immune infiltration in the normal group­(blue) and the liver cirrhosis group­(red). (C) Correlation heatmap displaying interrelationships among immune cell populations. (D) Correlation between mito-DEGs and immune cells. *p < 0.05; **p < 0.01; ***p < 0.001; ****p < 0.0001.

### 3.4 Identify Hub Genes of Mito-DEGs by WGCNA

In the LC data set, WGCNA identified an optimal soft-thresholding power of 13 (Figure 5A). A total of six modules were identified, with the red module comprising 743 genes strongly correlated with liver cirrhosis (Figure 5B). Furthermore, a scatter plot revealed a positive association (*r* = 0.59, *p* < 0.001) across the red module membership and gene importance for liver cirrhosis (Figure 5C). As consequently, the red module was regarded as the crucial module for next analysis. Venn analysis revealed RAB7B and NRAS at the mitophagy-module intersection (Figure 5D).

**Figure 5:**
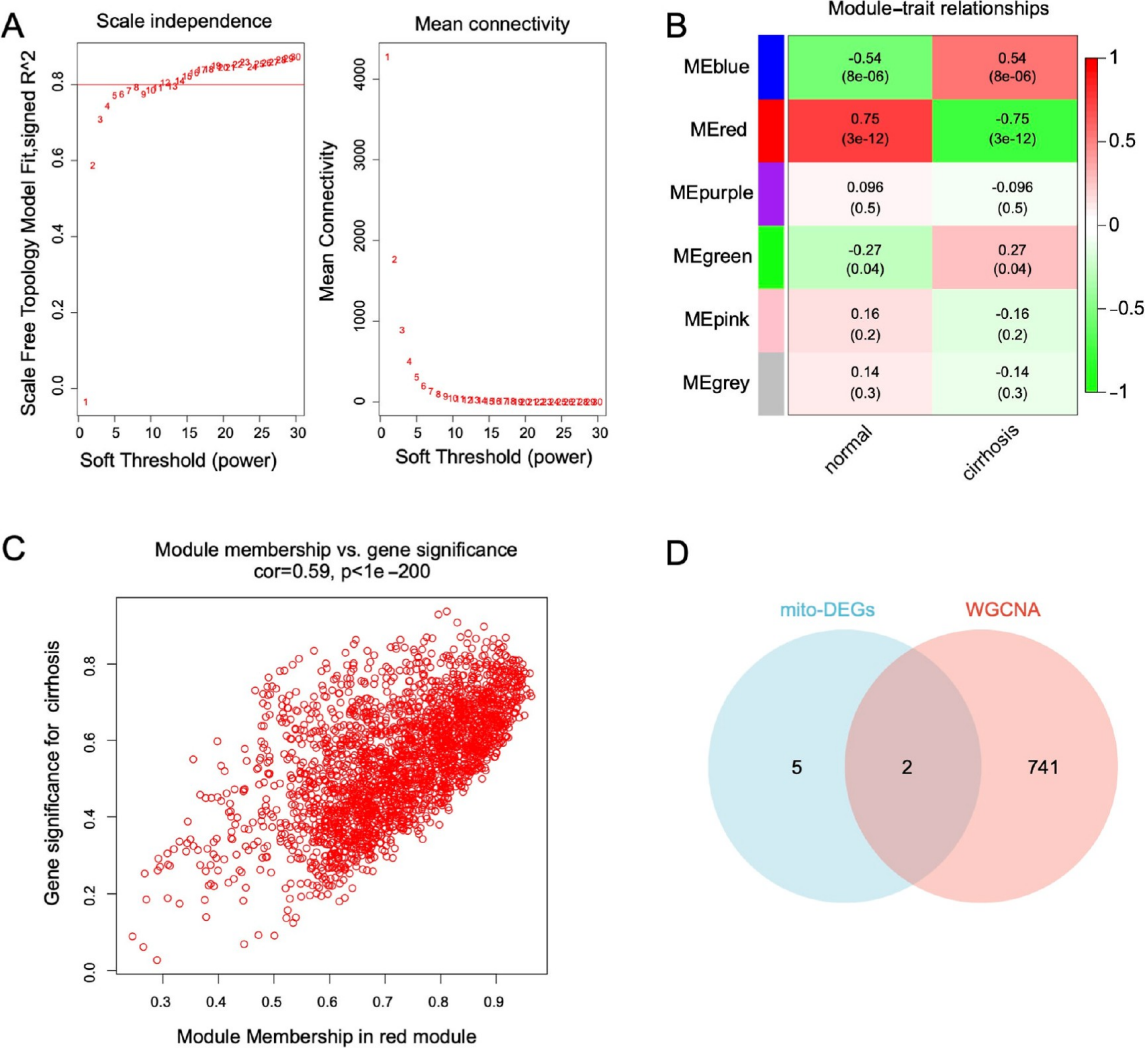
Identification of hub genes among mito-DEGs through WGCNA. (A) Selection of the optimal soft thresholding power. (B) Heatmap showing the correlation between six modules. (C) Scatter plot illustrating the correlation between the module membership of the red module and gene significance for liver cirrhosis. (D) Venn diagram showing the shared genes between mito-DEGs and the red module.

### 3.5 External Validation of Hub Genes

To identify and screen candidate genes associated with liver fibrosis development, we employed microarray data sets from public databases. The analysis of the GSE25097 data set indicated higher expression levels of RAB7B and NRAS in cirrhotic livers compared to normal livers (Figure 6A). In addition, the GSE84044 data set indicated increased expression of RAB7B and NRAS in patients with significant fibrosis (Scheuer score S2–4) than those without significant fibrosis (Scheuer score S0–1) (Figure 6B). Notably, RAB7B exhibited consistent upregulation across the LC data set, GSE25097 and GSE84044, whereas NRAS showed a divergent regulation. Additionally, we performed a correlation analysis between RAB7B, NARS, and key liver fibrosis markers. The findings revealed a significant positive correlation (*r* = 0.739 and 0.468, *p* < 0.001 and *p* < 0.001, respectively) between RAB7B and collagen alpha 1 (COL1α1) (Figure 6C, D), while NRAS exhibited a weaker association with COL1α1 (*r* = 0.604 and 0.418, *p* < 0.001 and *p* < 0.001, respectively) (Figure 6C, D). Based on these findings, we selected RAB7B for further investigation.

**Figure 6:**
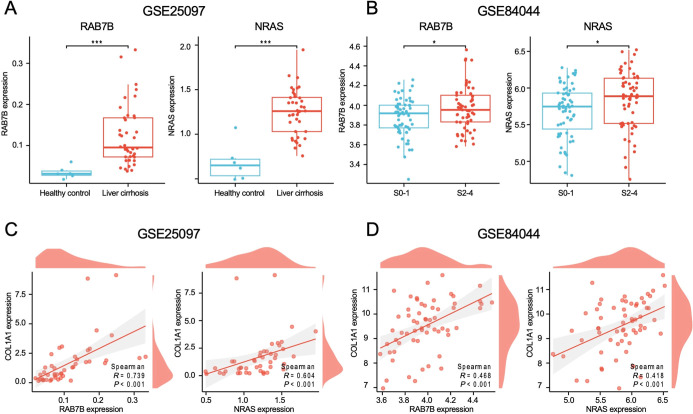
External validation of hub genes. (A,B) Gene expression levels in the validation data sets GSE25097 (A) and GSE84044 (B). (C,D) Correlation between hub genes and COL1α1. Values are represented as mean ± SD. Student *t*-test (A,B). Spearman’s correlation (C,D). *p < 0.05; **p < 0.01; ***p < 0.001; ****p < 0.0001, n.s., not significant.

### 3.6 Validation of RAB7B Expression in HSCs

According to the bioinformatic analyses above, RAB7B could have a role in the process of liver cirrhosis. The cell types that express RAB7B in the liver were identified using the Human Protein Atlas. The findings showed that HSCs, cholangiocytes, and plasma cells were the primary locations for RAB7B expression in the liver (Figure 7A). In addition, we found that RAB7B is predominantly localized in the cell population of myofibroblasts with Stmn1 expression, as revealed by single-cell RNA sequencing analysis of liver tissues from carbon tetrachloride-induced mouse models (Figure 7B–D). Given the pivotal role of HSCs activation in liver cirrhosis, we investigated RAB7B expression dynamics during HSCs activation. After extensive research, it was shown that the human HSC-derived LX-2 cell line had profibrotic characteristics.^28^ We employed to assess RAB7B regulation under activation conditions. The findings demonstrated that when HSCs are cultivated in DMEM supplemented with 10% fetal bovine serum (FBS), they display a pro-fibrotic phenotype.^29^ We then evaluated RAB7B expression using a model that was 10% FBS-stimulated. After 24 h of incubation with 10% FBS, we found that LX-2 cells expressed more RAB7B and COL1α1 than cells grown in a control condition with 2% FBS (Figure 7E,F). In an LX-2 cell activation model induced by transforming growth factor-β (TGF-β), RAB7B mRNA levels were markedly increased at 24- and 48 h postexposure. (Figure 7G). In addition, COL1α1 was significantly upregulated following TGF-β stimulation (Figure 7G). Western blot also confirmed RAB7B protein elevation (Figure 7H). Collectively, these data establish RAB7B upregulation as a hallmark of HSC activation.

**Figure 7:**
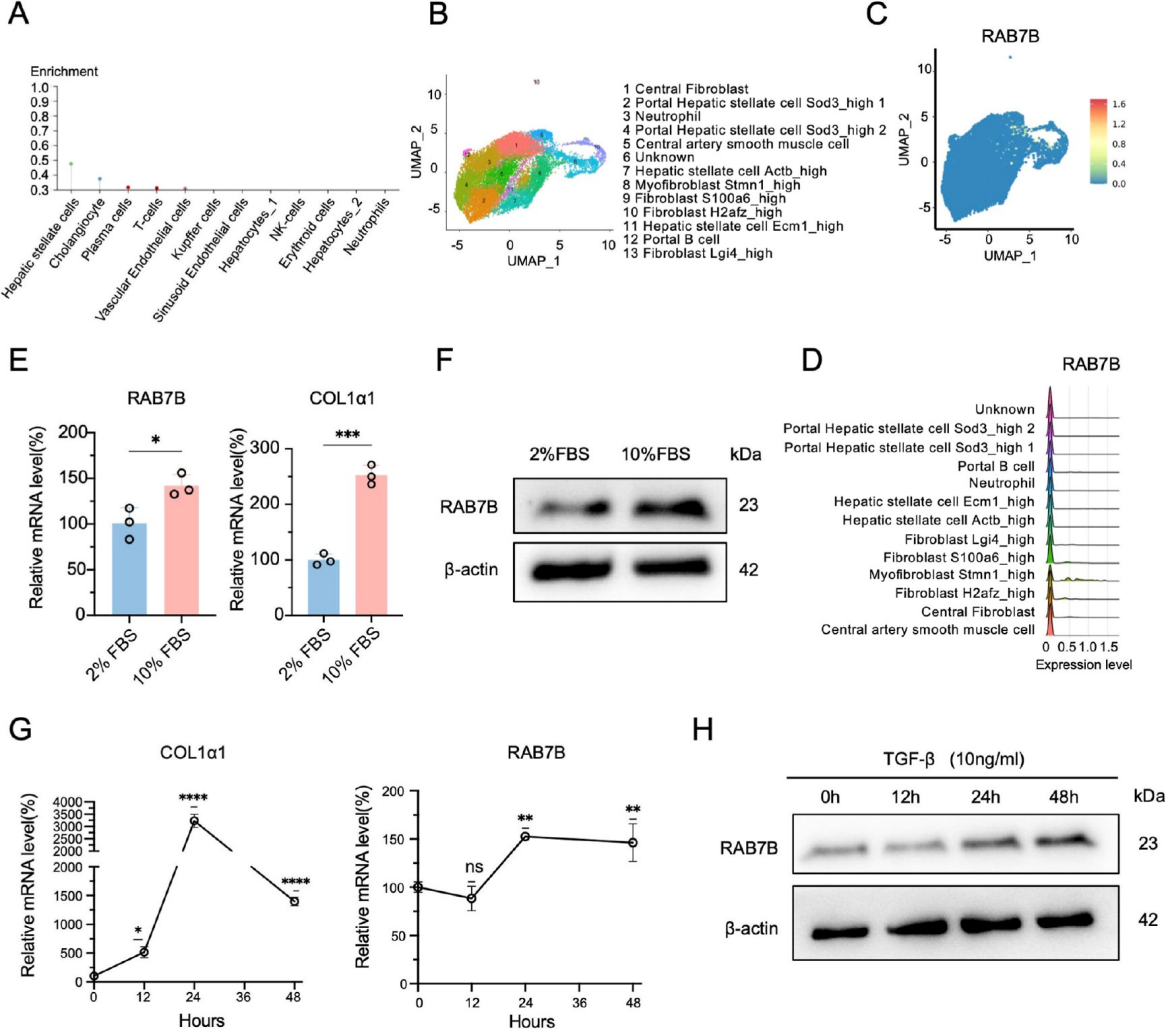
Validation of RAB7B expression in HSCs. (A) Cell types predominantly expressing RAB7B in the liver, as retrieved from the Human Protein Atlas. (B) Identification and annotation of major cell types. (C) RAB7B expression in different cell clusters. (D) Ridge plot of RAB7B expression in different cell types. (E) qRT-PCR and (F) Western blot of RAB7B and COL1α1 expression in LX-2 cells cultured in 2% FBS and 10% FBS for 24 h. (G) RAB7B and COL1α1 mRNA expression in LX-2 cells at different time points (0 h, 12 h, 24 h, 48 h) after TGF-β treatment. (H) Western blot of RAB7B in LX-2 cells at different time points (0 h, 12 h, 24 h, 48 h) of TGF-β treatment. Values are represented as mean ± SD. Student *t*-test (E). Ordinary one-way ANOVA (G). *p < 0.05; **p < 0.01; ***p < 0.001; ****p < 0.0001, n.s., not significant.

### 3.7 Validation of RAB7B Expression in CCL_4_-Induced Liver Cirrhosis Mice

To further validate the results obtained from the previous data set, we induced liver cirrhosis using CCl_4_ in mice. Sirius red, Masson, and α-SMA staining demonstrated significant collagen fiber deposition around the hepatic lobules and within the confluent region of liver cirrhosis mice (Figure 8A). Immunohistochemistry analysis further confirmed that RAB7B expression was markedly upregulated in CCl_4_-induced cirrhotic livers (Figure 8A). To validate the expression of RAB7B in liver cirrhosis, we quantified mRNA levels in liver specimens from CCl_4_-induced cirrhotic mice. The findings showed that liver cirrhosis was associated with elevated expression of COL1α1, an activation marker for HSCs, and persistently higher levels of RAB7B expression than normal control mice. (Figure 8B). Moreover, correlation analysis revealed a positive relationship between RAB7B and COL1α1 expression (*r* = 0.502, *p* = 0.030) in CCl_4_-treated mice (Figure 8C). According to Western blot, cirrhotic livers have higher levels of RAB7B protein expression than normal liver tissues. (Figure 8D). These findings collectively demonstrate that RAB7B expression was upregulated in cirrhotic livers, providing strong evidence of a substantial association with liver cirrhosis.

**Figure 8:**
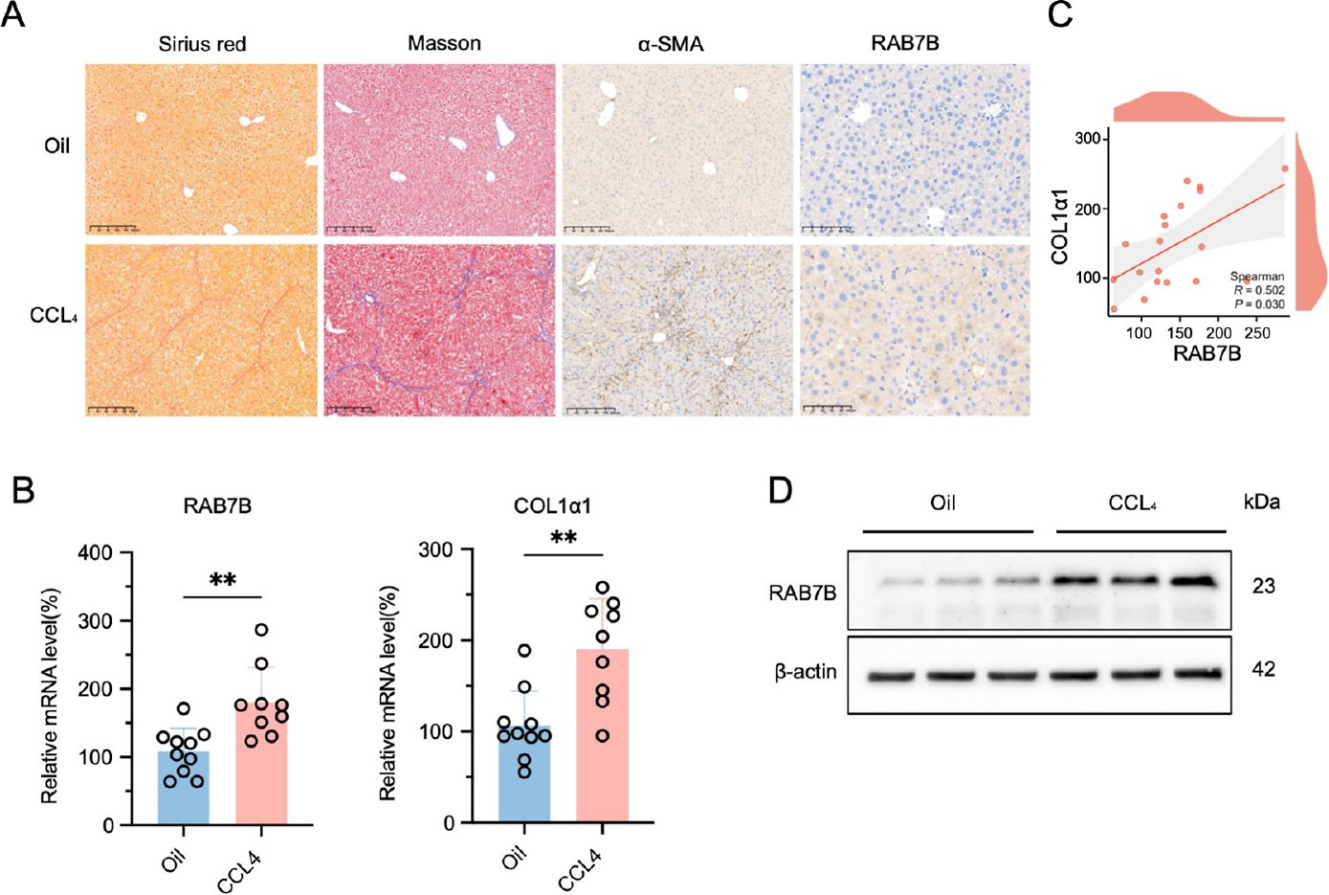
Validation of RAB7B expression in CCL4-induced liver cirrhosis mice. (A) Representative Sirius red, Masson, α-SMA, and RAB7B staining images in CCl_4_-induced fibrotic livers of mice (scale bars = 200 μm). (B) RAB7B and COL1α1 mRNA expression in CCl_4_-induced fibrotic livers of mice. (C) Correlation analysis of RAB7B mRNA expression with COL1α1 mRNA expression. (D) Western blot of RAB7B in CCl_4_-induced fibrotic livers of mice revealed by immunoblotting. Values are represented as mean ± SD. Student *t*-test (B). Spearman’s correlation­(C). *p < 0.05; **p < 0.01; ***p < 0.001; ****p < 0.0001, n.s., not significant.

### 3.8 Inhibition of RAB7B Reduces HSCs Activation In Vitro

Building on established evidence of RAB7B upregulation in activated HSCs, our hypothesis was that the activation phenotype of HSCs would be suppressed by RAB7B expression. To test this hypothesis, Western blot and qRT-PCR were used to confirm siRNA-mediated RAB7B knockdown. The results exhibited that si-RAB7B had a significant interference efficiency as evidenced by a reduction in RAB7B mRNA and protein levels (Figure 9A,B). It has been demonstrated that activated HSCs exhibit altered phenotypes, including migration toward the site of injury, increased cell proliferation, and expression of α-SMA.^30,31^ RAB7B knockdown impaired migration in activated LX-2 cells according to the transwell experiment. TGF-β stimulation increased LX-2 cell migration by 3.0-fold, which was attenuated by 63% with RAB7B silencing (Figure 9C,D). EdU proliferation assays demonstrated that RAB7B knockdown reduced TGF-β induced LX-2 cell proliferation by 45% (Figure 9C,E). Furthermore, immunofluorescence analysis revealed that inhibition of RAB7B effectively suppressed the expression of α-SMA (Figure 9C,F). Notably, RAB7B silencing abrogated TGF-β mediated COL1α1 upregulation, showing 61% suppression compared to stimulated controls (Figure 9G).

**Figure 9:**
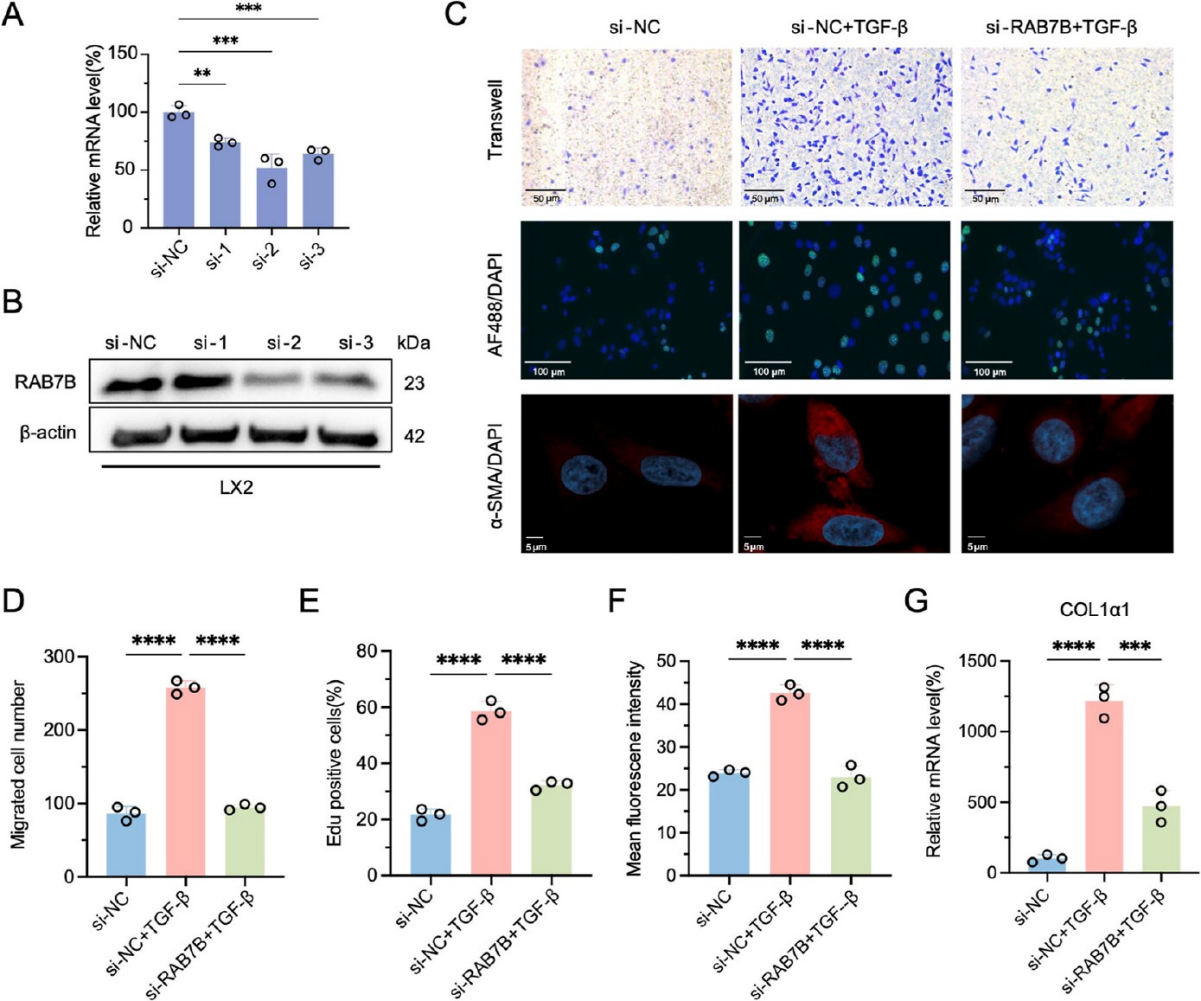
Inhibition of RAB7B reduces HSCs activation in vitro. (A) qRT-PCR and (B) Western blot of RAB7B knockdown by siRNA in LX-2 cells. (C) Representative images s of cell migration (upper panels, scale bar = 50 μm), EdU proliferation (middle panels, scale bar = 100 μm), and α-SMA immunofluorescence staining (bottom panels, scale bar = 5 μm) in RAB7B knockdown LX-2 cells and negative control (si-NC) cells, with or without TGF-β treatment. (D) Quantitative analysis of migrated cell number. (E) Quantitative analysis of Edu positive cells. (F) Quantitative analysis of mean fluorescence intensity about α-SMA. (G) COL1α1 mRNA expression in RAB7B knockdown LX-2 cells and si-NC-transfected LX-2 cells treated with or without TGF-β. Values are represented as mean ± SD. Ordinary one-way ANOVA (A,D–G). *p < 0.05; **p < 0.01; ***p < 0.001; ****p < 0.0001, n.s., not significant.

### 3.9 Identification of Candidate Therapeutic Agents for the Treatment of Liver Cirrhosis

A total of 150 upregulated and 150 downregulated DEGs were submitted to the CMap database to screen for potential small-molecule therapeutics for liver cirrhosis. Through this analysis, six candidate drugs with high connectivity scores were discovered, including mofezolac, parbendazole, *N*-formylmethionylalanine, SKF-89976A, LDN-193189, and celastrol (Figure 10A). The structures of the identified compounds were retrieved from the PubChem database and shown in Figure 10B–G.

**Figure 10:**
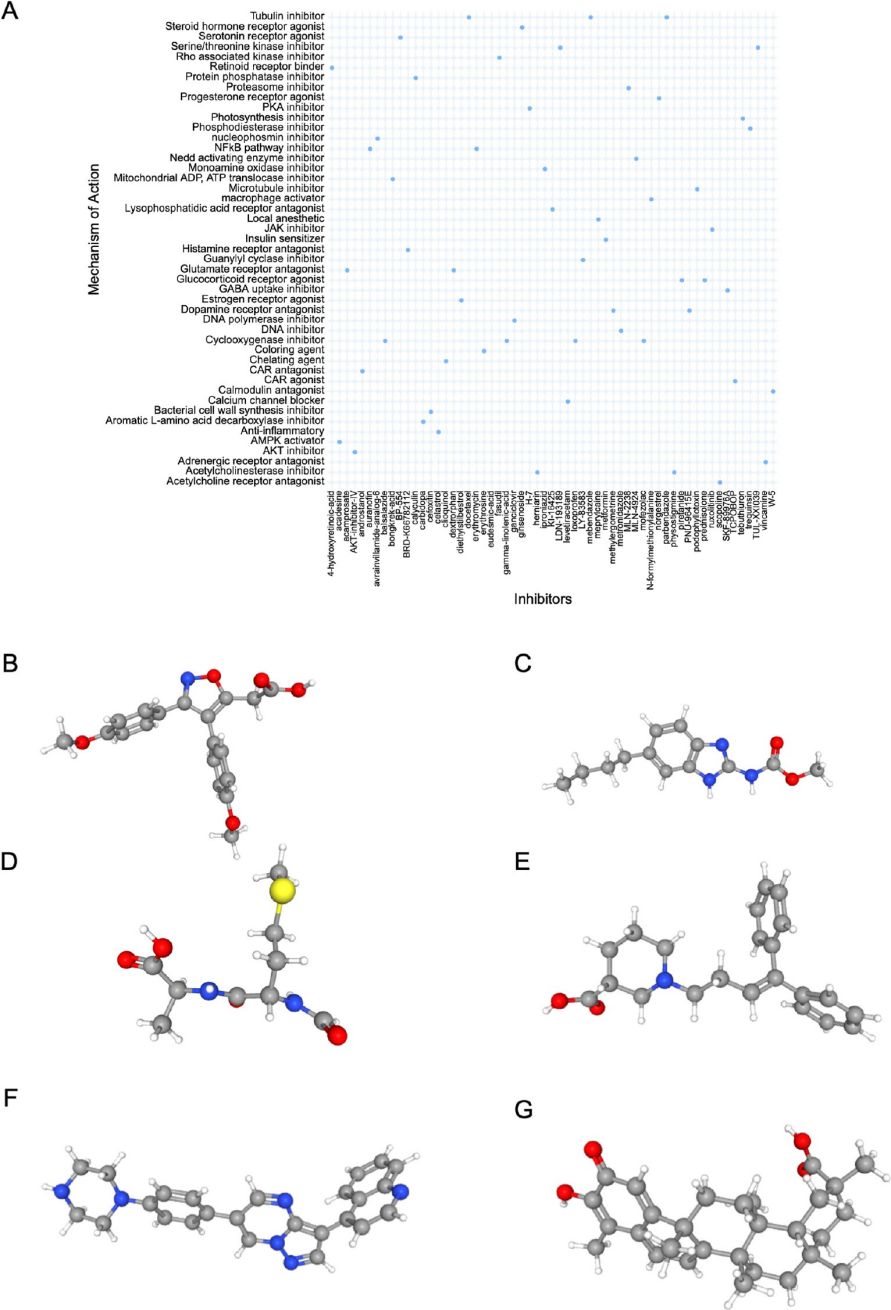
Prediction of potential therapeutic drugs. (A) CMap analysis of the small-molecule compounds. (B–G) 3D structures of small molecule compounds predicted using the PubChem database, including (B) mofezolac, (C) parbendazole, (D) *N*-formylmethionylalanine, (E) SKF-89976A, (F) LDN-193189 and (G) celastrol.

### 3.10 The Molecular Docking Analysis of RAB7B

Molecular docking analysis was performed to identify potential interactions between RAB7B and selected therapeutic compounds. The calculated binding energies for the RAB7B-drug complexes were as follows: mofezolac (−6.4 kcal/mol, Figure 11A), parbendazole (−7.0 kcal/mol, Figure 11B), *N*-formylmethionylalanine (−5.3 kcal/mol, Figure 11C), SKF-89976A (−6.8 kcal/mol, Figure 11D), LDN-193189 (−9.1 kcal/mol, Figure 11E), and celastrol (−8.6 kcal/mol, Figure 11F). These results indicate strong molecular interactions between RAB7B and the six candidate compounds.

**Figure 11:**
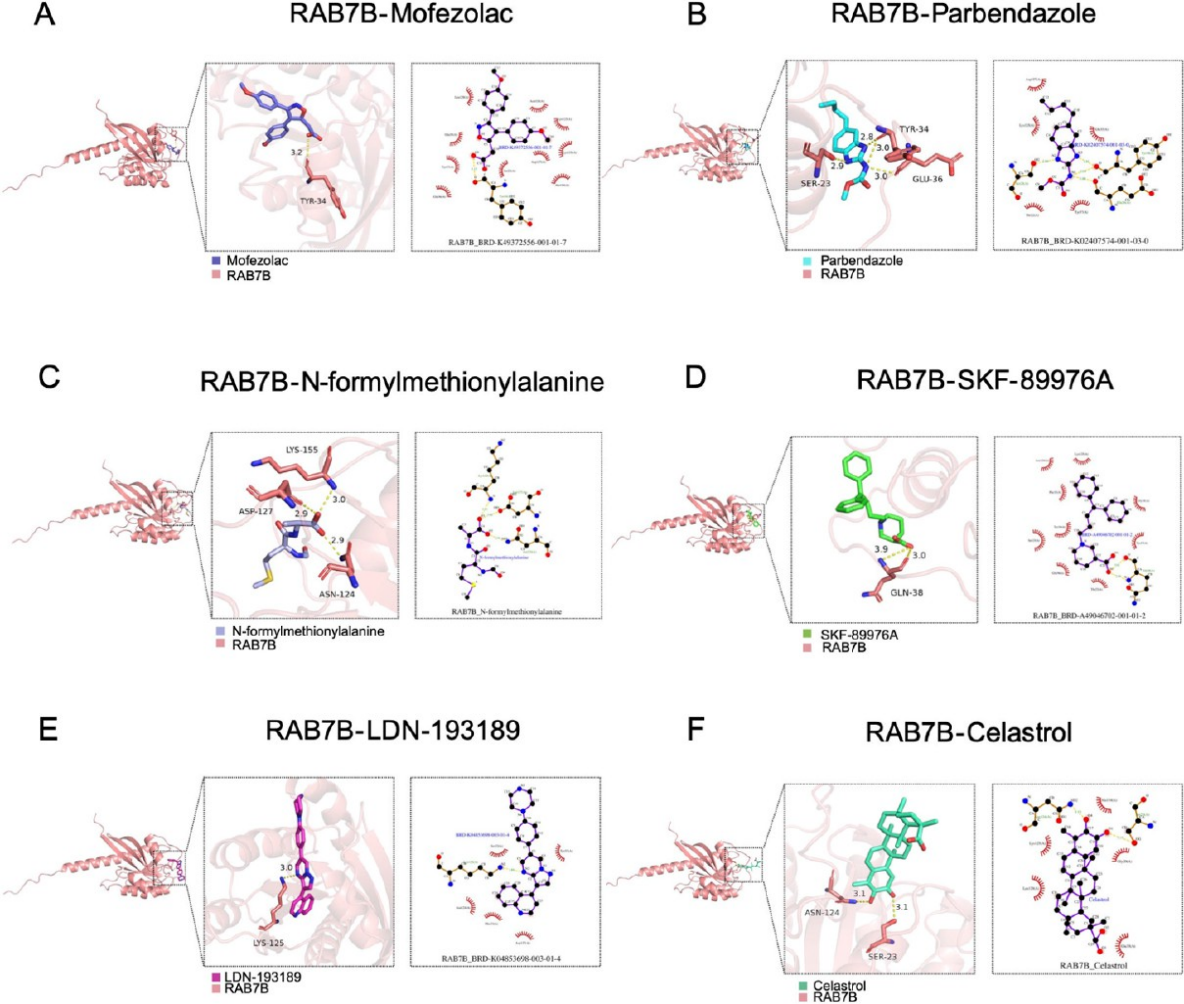
Molecular docking analysis. Molecular docking results for (A) RAB7B–mofezolac complex, (B) RAB7B–parbendazole complex, (C) RAB7B–*N*-formylmethionylalanine complex, (D) RAB7B–SKF-89976A complex, (E) RAB7B–LDN-193189 complex, and (F) RAB7B–celastrol complex.

### 3.11 RAB7B Deficiency Enhances Mitophagy under Fibrogenic Stress

To further clarify the role of RAB7B in mitophagy, we performed functional and regulatory analyses in LX2 cells. TGF-β stimulation induced p62 accumulation, LC3-II reduction, TOMM20 elevation, and decreased PINK1 and Parkin, indicating impaired mitophagy. Notably, RAB7B knockdown reversed these alterations, with reduced p62 and TOMM20 and increased LC3-II, PINK1, and Parkin, suggesting that RAB7B deficiency promotes mitophagy and prevents mitochondrial accumulation (Figure 12A,B). Consistently, MitoTracker and LysoTracker staining showed enhanced colocalization of mitochondria and lysosomes after RAB7B knockdown, further supporting its inhibitory role in mitophagy (Figure 12C). In addition, we examined RAB7B expression under altered mitophagy states. Chloroquine (CQ)-mediated mitophagy inhibition led to elevated p62, LC3-II, TOMM20, and RAB7B, whereas CCCP-induced mitophagy activation decreased p62, TOMM20, and RAB7B while increasing LC3-II (Figure 12D–G). Together, these findings indicate that RAB7B negatively regulates mitophagy and that its expression is dynamically modulated by mitophagy activity.

**Figure 12:**
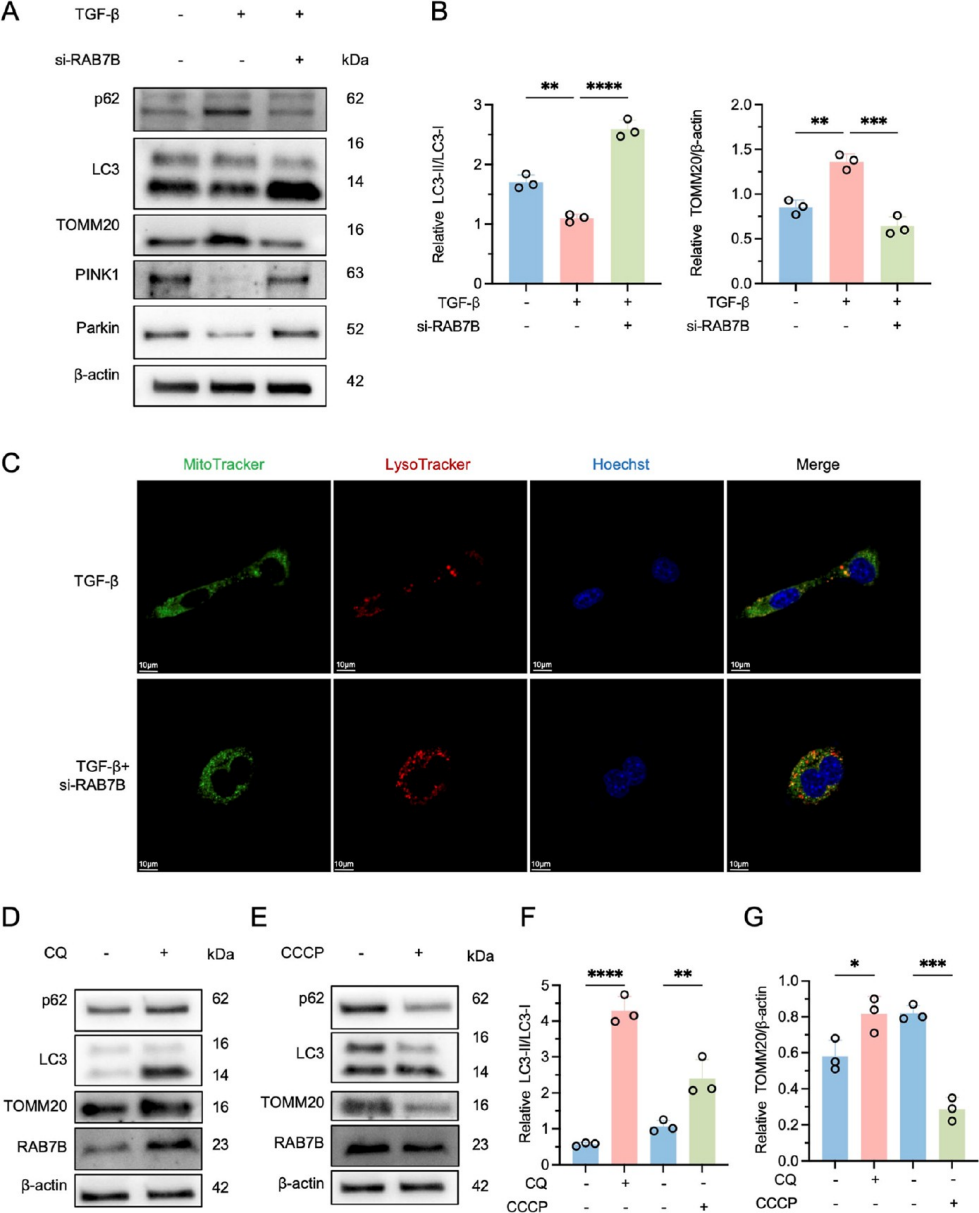
RAB7B deficiency enhances mitophagy under fibrogenic stress. (A) Western blot analysis of mitophagy markers in LX2 cells treated with TGF-β, with or without RAB7B knockdown. (B) Quantification analysis of LC3-II/LC3-I ratios and TOMM20. (C) Representative fluorescence images of MitoTracker and LysoTracker staining in LX2 cells (scale bars = 10 μm). (D,E) Western blot analysis of LX2 cells treated with CQ or CCCP. (F,G) Quantification analysis of LC3-II/LC3-I ratios and TOMM20. Values are represented as mean ± SD. Ordinary one-way ANOVA (B,F,G). *p < 0.05; **p < 0.01; ***p < 0.001; ****p < 0.0001, n.s., not significant.

## 4 Discussion

Liver cirrhosis represents a chronic hepatic disorder arising from sustained liver injury, marked by excessive extracellular matrix accumulation and destruction of normal liver structure and function. Disease progression culminates in decompensated liver failure and hepatocarcinogenesis, conditions associated with elevated mortality and limited therapeutic options, constituting a major global health burden.^32^ Mitophagy, a form of selective autophagy, specifically eliminates dysfunctional mitochondria through lysosomal degradation.^33^ It is essential for minimizing cellular damage and maintaining intracellular mitochondrial quality. Emerging evidence identifies mitophagy dysregulation as a pathogenic determinant in cirrhosis progression, with particular pathophysiological relevance to disease advancement. However, studies on mitophagy in liver cirrhosis have primarily focused on parenchymal hepatocytes, while research on its effects on HSCs and liver fibrosis remains limited. In this study, we sought to bridge this gap by investigating the mitophagy gene RAB7B, which is predominantly localized in HSCs, and assessing its role in HSCs activation. Our results suggest that RAB7B possesses strong predictive capabilities for the development of liver cirrhosis. Moreover, RAB7B expression was consistently elevated in both internal and external data sets, with a significant positive correlation to COL1α1 expression in cirrhotic patients compared with controls. Notably, RAB7B expression was particularly high in HSCs. In vitro experiments revealed that RAB7B promotes HSCs activation, migration, and proliferation, and inhibiting RAB7B can attenuate these processes. Our findings uncover a mechanistic connection between HSCs mitophagy and cirrhotic pathogenesis, positioning RAB7B as a novel druggable target for antifibrotic therapies.

Our integrative analysis of the LC data set identified 2101 DEGs including 1044 downregulated and 1105 upregulated genes. According to functional enrichment, these DEGs were discovered to be primarily enriched in fatty acid metabolism, mitochondrial matrix, electron transport chain activity, and oxidoreductase, which are the key mechanisms involved in liver cirrhosis development. Mitochondrial functional integrity is essential for maintaining liver bioenergetics. Structural and functional dysfunction of mitochondria, including damage to the electron transport chain and overproduction of free radicals, can lead to reduced fatty acid β-oxidation. It will exacerbate lipid accumulation and injury in hepatocytes, leading to cirrhosis.^34^ To elucidate the effect of mitophagy on cirrhosis, we identified seven candidate mito-DEGs by intersecting the mitophagy gene sets with the DEGs, all of which are potential predictors of cirrhosis development. Furthermore, we used the CIBERSORT and discovered a substantial correlation between cirrhosis, memory B cells, and regulatory T cells. We used WGCNA to perform the LC data set to further discover modular genes that are substantially linked with cirrhosis. Based on this method, we identified the most significant “red module” containing 743 genes and performed subsequent intersection analysis with mito DEGs. This intersection yielded two hub genes: RAB7B and NRAS. Among them, RAB7B demonstrated stronger expression enrichment in cirrhosis and stronger correlation with COL1α1 expression, thus prioritized for downstream validation. Deng also discovered that RAB7B expression was upregulated in dysfunction-associated steatohepatitis with diagnostic potential.^35^ Additionally, we explored the biological function of RAB7B and found that it was primarily localized in HSCs in the liver, as indicated by the HPA database and single-cell RNA sequencing. HSCs play a crucial role in liver cirrhosis, differentiating into myofibroblasts and producing vast amounts of extracellular matrix, driving fibrosis formation. Thus, Pharmacological targeting of HSC activation remains a cornerstone of antifibrotic therapy development.^36^ Zhou reported that IGF2BP2 was elevated in activated HSCs and silencing IGF2BP2 could inhibit HSCs activation and liver fibrosis.^37^ In this study, RAB7B was found to be significantly upregulated in 10% serum and TGF-β induced environments, and its expression in the liver of cirrhotic mice was notably higher compared to controls. Finally, transwell assay, Edu staining, and immunofluorescence revealed that interfering with RAB7B in HSCs significantly inhibited their activation, migration, and proliferation. RAB7B showed notable binding interactions with the candidate compounds identified for treating liver cirrhosis. Surprisingly, no previous studies have specifically addressed the effects of RAB7B on cirrhosis or its specific role in HSCs. Therefore, these findings establish RAB7B as a dual-function biomarker and therapeutic target for liver cirrhosis management.

RAB7B, a small GTPase family member, is predominantly found in late endosomes and lysosomes. RAB7B was initially found in dendritic cells (DCs) and is expressed during monocyte and megakaryocyte differentiation.^38^ RAB7B modulates autophagy flux through interaction with ATG4B and regulates myosin II activation via coupling with TRPML1 Ca^2+^ channels, coordinating both cellular motility and lysosomal function.^38–40^ Additionally, RAB7B negatively modulates toll-like receptor (TLR) signaling via lysosomal degradation of TLR4, thereby attenuating inflammatory responses.^41–43^ RAB7B suppresses oral squamous cell carcinoma cell proliferation by autophagy modulation.^44^ However, no study has systematically explored the function of RAB7B in liver cirrhosis and the potential of RAB7B as a therapeutic target, and this study aims to address this gap.

In this study, we identified RAB7B as a mitophagy-related hub gene and validated its functional role in hepatic stellate cells. TGF-β stimulation impaired mitophagy, whereas RAB7B knockdown reversed these effects, indicating that RAB7B deficiency promotes mitochondrial clearance. Furthermore, RAB7B knockdown enhanced mitochondria-lysosome colocalization, providing direct evidence of its involvement in the mitophagy pathway. These findings align with previous reports showing that RAB7B interacts with Atg4B to regulate LC3 processing and autophagic flux, supporting its role as a negative regulator of autophagy.^40^ Importantly, we also found that RAB7B expression is dynamically modulated by mitophagy status. It is upregulated upon mitophagy inhibition and downregulated during mitophagy activation. Together, these results suggest that RAB7B is not only a bioinformatically predicted gene but also a functional and responsive regulator of mitophagy. Dysregulation of RAB7B may impair mitochondrial clearance, promote mitochondrial accumulation, and thereby contribute to the progression of liver cirrhosis.

Next, although our molecular docking analysis suggested strong interactions between RAB7B and several candidate compounds, it is important to note the translational challenges. The druggability of RAB7B is not yet established, and the high structural similarity among RAB GTPases may raise concerns of specificity and off-target effects. Furthermore, compounds such as celastrol have reported adverse effects in preclinical models (e.g., hepatotoxicity, gastrointestinal inflammation, neurotoxic effects.), highlighting the need for careful evaluation.^45,46^ Thus, while our findings provide preliminary insights, further preclinical studies are necessary to assess pharmacokinetics, safety, and therapeutic efficacy in vivo.

In addition, this study still has several limitations. Despite being sourced from public databases, the limited sample size may affect the accuracy and reliability of the result. Furthermore, the analysis was based on bioinformatics techniques and lacked raw sequencing data, which requires more validation before clinical application. Lastly, additional ex vivo and in vivo studies are necessary to completely understand the molecular process and mechanisms of RAB7B in liver cirrhosis.

## 5 Conclusions

Ultimately, our comprehensive bioinformatics study established mito-DEGs as key regulators in cirrhotic pathogenesis. We further verified the increased expression of RAB7B in an animal model of liver cirrhosis and uncovered the pivotal regulatory role of RAB7B in HSCs activation. Our findings suggest prospective therapeutic strategies for the therapy of cirrhotic patients by specifically targeting these mito-DEGs, particularly RAB7B.

## Supplementary Material



## Data Availability

The data sets analyzed during the current study are available in the GEO repository at https://www.ncbi.nlm.nih.gov/geo/ (accession number: GSE77627, GSE139602, GSE137720, GSE25097, and GSE84044).

## Notes

Data collection and preprocessing; the list of mitophagy-related genes; target sequences used for Rab7b knockdown; primer sequences list; antibodies used for Western blot and immunofluorescence (PDF)
