# Supplementary material for: RAB7B as a Potential Therapeutic Target in Liver Cirrhosis: Insights from Protein Expression and Bioinformatics Analyses
Source: ACS Omega. 2025 Dec 1;10(49):60491–507. doi: 10.1021/acsomega.5c08027 (PMC12713460; doi:10.1021/acsomega.5c08027)
Supplement: Supplementary file 1 [file ao5c08027_si_001.pdf]

Cover Page for Supporting Information

Manuscript Title:

RAB7B as a Potential Therapeutic Target in Liver Cirrhosis: Insights from Protein Expression and Bioinformatics Analyses

Authors:

Jinyao Dai[1#], Shuaibing Ying[1#], Jie Lin[2#], Yong Pan[1], Qiudan Zhang[1], Jing Liu[1], Yan Lou[1]*, Yunqing Qiu[1]*

Affiliations:

1 State Key Laboratory for Diagnosis and Treatment of Infectious Diseases, National Clinical Research Center for Infectious Diseases, National Medical Center for Infectious Diseases, Collaborative Innovation Center for Diagnosis and Treatment of Infectious Disease, Zhejiang Provincial Key Laboratory for Drug Evaluation and Clinical Research of Zhejiang Province, The First Affiliated Hospital, Zhejiang University School of Medicine, Hangzhou, 310003, China.

2 Department of General Surgery, Sir Run-Run Shaw Hospital, Zhejiang University School of Medicine, Hangzhou, 310003, China.

[#] Authors contributed equally

Contents:

(1) Data collection and pre-processing. **Fig. S1**

(2) The list of mitophagy-related genes. **Table S1**

(3) Target sequences used for Rab7b knockdown. **Table S2**

(4) Primer sequences list. **Table S3**

(5) Antibodies used for western blot and immunofluorescence. **Table S4**

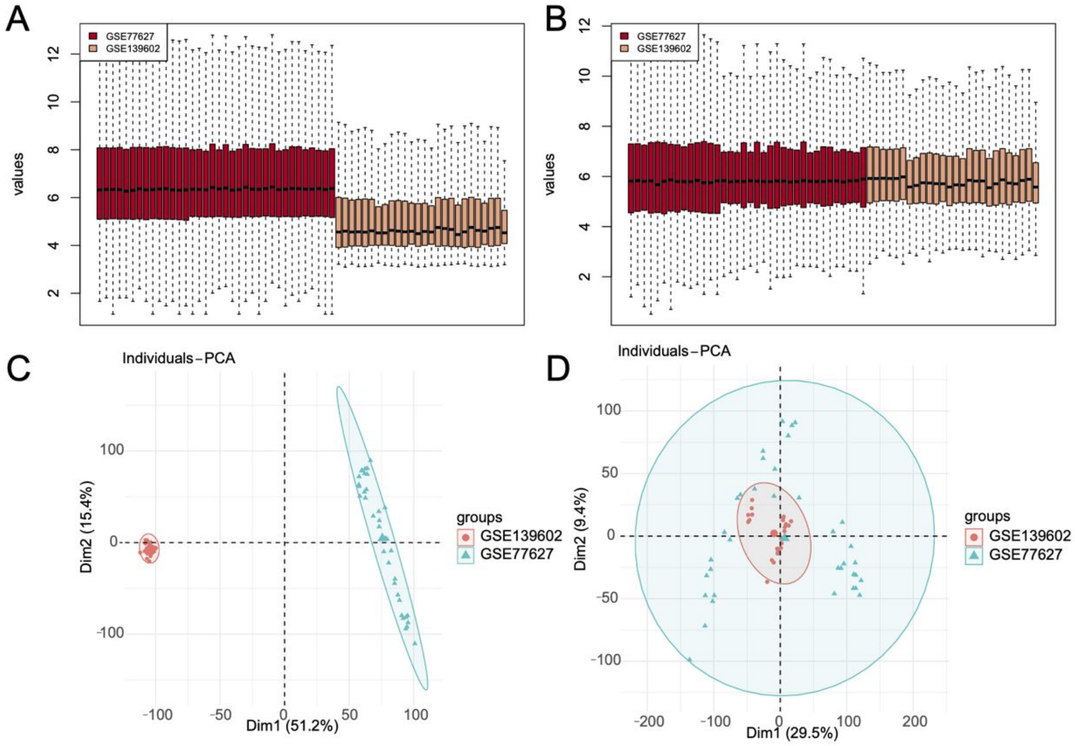

Fig. S1. Data collection and pre-processing.

Box plot of the LC dataset (A) before correction and (B) after correction. Principal component analysis (PCA) plot of the LC dataset (C) before correction and (D) after correction.

Table S1 The list of mitophagy-related genes

id	Gene symbol	id	Gene symbol	id	Gene symbol
1	ATG12	26	SRC	51	TFEB
2	ATG5	27	CSNK2A1	52	TFE3
3	MFN1	28	CSNK2A2	53	BECN
4	MTERF3	29	CSNK2B	54	BCL2L1
5	SQSTM1	30	BCL2L13	55	CSNK2A
6	VDAC1	31	EIF2AK3	56	HIF1A
7	MFN2	32	ATF4	57	E2F1
8	MAP1LC3A	33	JNK	58	RELA
9	MAP1LC3B	34	JUN	59	BNIP3

10	TOMM40	35	TOM7	60	HRAS
11	TOMM70	36	PARK2	61	KRAS
12	TOMM22	37	RHOT1	62	NRAS
13	TOMM20	38	RHOT2	63	MRAS
14	TOMM6	39	USP8	64	RRAS
15	TOMM7	40	USP15	65	RRAS2
16	TOMM5	41	USP30	66	TP53
17	PINK1	42	TAX1BP1	67	BNIP3L
18	PRKN	43	CALCOCO2	68	FOXO3
19	UBC	44	OPTN	69	CITED2
20	UBB	45	NBR1	70	SP1
21	UBA52	46	TBK1	71	TBC1D15
22	RPS27A	47	GABARAP	72	TBC1D17
23	ULK1	48	AMBRA1	73	RAB7A
24	PGAM5	49	ATG9	74	RAB7B
25	FUNDC1	50	MITF	75	FIS1

Table S2 Target sequences used for Rab7b knockdown.

siRNA	Primer sequences
si-RAB7B-1	Sense 5’-CUCAUUAUCGUCGGAGCCAUUdTdT-3’
	Antisense 5’-AAUGGCUCCGACGAUAAUGAGdTdT-3’
si-RAB7B-2	Sense 5’-AGUGCCAAGAAUGACAUCAAUdTdT-3’
	Antisense 5’-AUUGAUGUCAUUCUUGGCACUdTdT-3’
si-RAB7B-3	Sense 5’-GCUGGUGUAGAGAGAAAGAUAdTdT-3’
	Antisense 5’-UAUCUUUCUCUCUACACCAGCdTdT-3’

Table S3 Primer sequences list.

Gene	Primer sequences

h-β-actin	Forward 5'- CTCTTCCAGCCTTCCTTCCT -3'
	Reverse 5'- AGCACTGTGTTGGCGTACAG -3'
h-Rab7b	Forward 5'-TGGTGTCCACGTTCTACAAGG-3'
	Reverse 5'-CCAGGGCTTCAAAAGACTCCA-3'
m-β-actin	Forward 5'- CCACCATGTACCCAGGCATT -3'
	Reverse 5'- CGGACTCATCGTACTCCTGC -3'
m-Rab7b	Forward 5'-CCCTATGGAGCAGTCATATCCC -3'
	Reverse 5'- TCAAAGGCTTGTACCACATTGAT-3'
h-COL1α1	Forward 5'- GTGCGATGACGTGATCTGTGA-3'
	Reverse 5'- CGGTGGTTTCTTGGTCGGT-3'
m-COL1α1	Forward 5'-GACAGGCGAACAAGGTGACAGAG-3'
	Reverse 5'-CAGGAGAACCAGGAGAACCAGGAG-3'

Table S4 Antibodies used for western blot and immunofluorescence.

Antibody	Company	Cat#	Host
Rab7b	Abcam	ab193360	R
α-SMA	Bioss	bs-10196R	R
LC3	CST	83506	M
P62	Proteintech	66184-1-Ig	M
TOMM20	HUABIO	ET1609-25	R
Parkin	HUABIO	HA722952	R
PINK1	HUABIO	ER1706-27	R
β-actin	Proteintech	HRP-66009	M
